# Triple Threat: Co-occurrence of Serous Borderline Ovarian Tumor, Endometrial Adenocarcinoma, and Low-Grade Appendiceal Mucinous Neoplasms With Pseudomyxoma Peritonei in a Single Patient

**DOI:** 10.7759/cureus.88343

**Published:** 2025-07-20

**Authors:** Ana Djordjevic, Aleksandar Rakic, Smiljana Donic, Nenad Pavlovic, Lazar Nejkovic

**Affiliations:** 1 Department of Obstetrics and Gynecology, Obstetrics and Gynecology Clinic Narodni Front, Belgrade, SRB; 2 Department of Pathology, Obstetrics and Gynecology Clinic Narodni Front, Belgrade, SRB

**Keywords:** endometrial cancer (ec), low-grade appendiceal mucinous neoplasm, pseudomyxoma peritonei, serous borderline ovarian tumor, synchronous neoplasms

## Abstract

Borderline ovarian tumors (BOTs) are epithelial ovarian neoplasms characterized by cellular proliferation and nuclear atypia without stromal invasion. On the other hand, low-grade appendiceal mucinous neoplasms (LAMNs) have features such as mucin production, a distinct invasion pattern, and low-grade cytological characteristics. Pseudomyxoma peritonei (PMP), an uncommon disorder characterized by mucinous peritoneal dispersion, can be exacerbated by both BOTs and LAMNs. We present a case of a 63-year-old patient who was diagnosed with a serous BOT, LAMN with PMP, and synchronous endometrioid endometrial cancer. After episodes of postmenopausal bleeding, an explorative curettage confirmed the diagnosis of endometrioid endometrial cancer. Since the preoperative ultrasound showed a tumorous mass in the right adnexa and retrouterine space, the patient had an explorative laparotomy. A right ovarian tumor, hepatic and omental deposits, and an appendiceal lesion were among the intraoperative findings. A hysterectomy was performed along with peritoneal biopsy, cytological analysis of peritoneal lavage, bilateral salpingo-oophorectomy, omentectomy, lymphadenectomy, and appendectomy. Histopathological examination verified the presence of BOT, LAMN with PMP, and endometrial cancer. This case highlights the diagnostic difficulties of synchronous gastrointestinal and gynecological neoplasms and emphasizes the significance of a multidisciplinary surgical approach and thorough preoperative examination. According to our knowledge, this is the first case of synchronous endometrial cancer, serous BOT, and LAMN with PMP.

## Introduction

Borderline ovarian tumors (BOTs) represent a group of epithelial ovarian neoplasms with distinct histological features. They are marked by increased cellular proliferation and mild nuclear atypia, yet they lack stromal invasion [1]. The incidence of BOT ranges from 1.8 to 4.8 cases per 100,000 women per year, and they comprise up to 20% of all ovarian epithelial neoplasms [2]. Almost 50% of BOTs have serous histological features [2]. Differentiating between benign, borderline, and malignant ovarian tumors based solely on imaging findings presents a significant challenge [3]. On the other hand, the low specificity of various tumor markers limits their diagnostic utility [4].

Low-grade appendiceal mucinous neoplasms (LAMNs) exhibit distinct low-grade cytologic characteristics, a specific invasion pattern through the appendiceal layers, and a villous or flat proliferative intestinal-type mucinous epithelium. These neoplasms are marked by substantial mucin production and notable constraints of the muscularis propria [5]. One of the important characteristics of BOTs and LAMN is that both can lead to a specific change called pseudomyxoma peritonei (PMP) [6,7].

Endometrial cancer presents the most common gynecological malignancy in developed countries, and its incidence continues to rise [8]. The occurrence of synchronous primary endometrial and ovarian carcinoma is relatively rare, which may lead to misinterpretation of endometrial cancer with ovarian metastasis [9]. On the other hand, there is limited knowledge regarding primary endometrial cancer and synchronous BOT. We report the first case of synchronous serous BOT, endometrial adenocarcinoma, and LAMN with PMP, highlighting diagnostic and management challenges.

## Case presentation

A 63-year-old patient, para three, gravida five, was referred to the Oncology Medical Advisory Board following histological analysis of samples obtained through exploratory curettage, which confirmed the diagnosis of endometrial adenocarcinoma. The intervention was conducted following events of irregular bleeding in postmenopause. In addition to presenting gynecological symptoms, the patient exhibited comorbidities including hypertension, type II diabetes mellitus, and chronic venous insufficiency. Upon admission to the clinic, an ultrasound examination revealed a tumorous mass in the right adnexa and retrouterine space. No additional pathological findings were observed on the ultrasound examination. The Oncology Medical Advisory Board has reached a consensus to proceed with an exploratory laparotomy.

During the surgical procedure, the team observed a cystic tumor characterized by a smooth capsule, which was found to be originating from the right ovary. Additionally, a tumor with a crumbly consistency was observed in the region of the right sacrouterine ligament, situated medially to the right ureter. Multiple round-shaped deposits were observed in the omentum, varying in size from several millimeters to 1 cm, along with two deposits located in the right lobe of the liver. The surgical team performed a hysterectomy with bilateral adnexectomy, interiliac lymphadenectomy, infracolic omentectomy, and appendectomy. An intraoperative frozen section (ex tempore) analysis was performed on the ovarian and omental specimens. The preliminary diagnosis included PMP and a serous BOT. Biopsies of the peritoneum were collected from the omentum and adjacent adipose tissue and demonstrated the presence of low-grade mucinous neoplastic epithelium. Cytological analysis of peritoneal lavage was conducted using 44 mL of ascitic fluid, revealing cytological smears with mucinous epithelial cells indicative of low-grade PMP.

**Figure 1:**
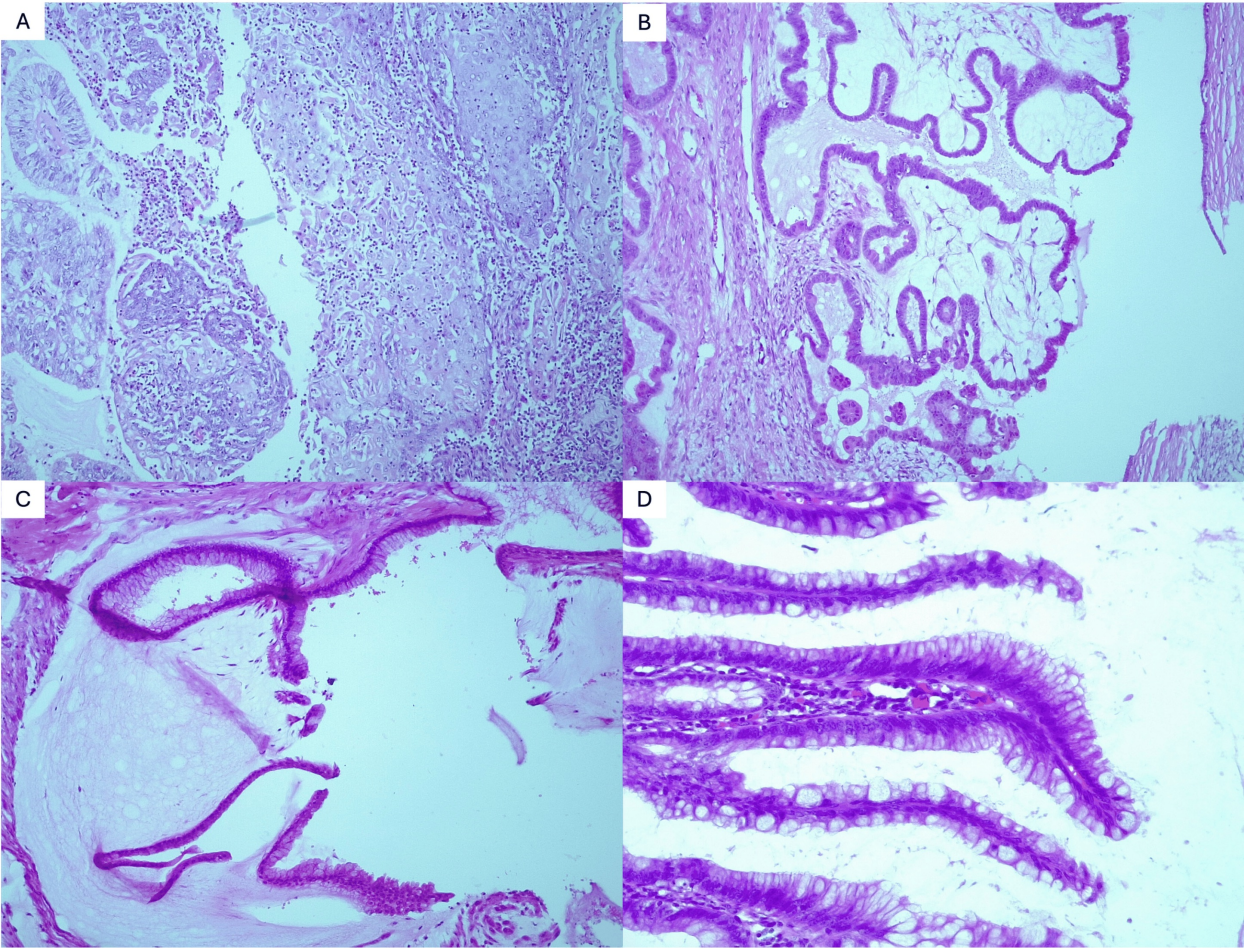
Histopathological (H&E) examination of the specimens after hysterectomy with billateral adnexectomy, omentectomy, and appendectomy. (A) Endometrioid endometrial adenocarcinoma with squamous differentiation (H&E, x5). (B) Serous borderline tumor in the right ovary (H&E, x10). (C) Pseudomyxoma peritonei in the omental sample (H&E, x20). (D) LAMN in the appendiceal sample (H&E, x20) H&E: hematoxylin & eosin; LAMN: low-grade appendiceal mucinous neoplasm

Histological examination revealed grade 1 endometrioid endometrial adenocarcinoma (Figure 1A), a serous BOT in the right ovary (Figure 1B), grade 1 PMP in the omental sample (Figure 1C) in the left ovary, alongside LAMN in the appendiceal specimen (Figure 1D). The final pathological staging was as follows: International Federation of Gynecology and Obstetrics (FIGO) stage IB for the endometrial adenocarcinoma (pT1bN0MxL0V0), FIGO stage IA for the BOT (pT1aN0Mx), and pT4aNxMxMic for the appendiceal low-grade mucinous neoplasm with peritoneal involvement.

**Figure 2:**
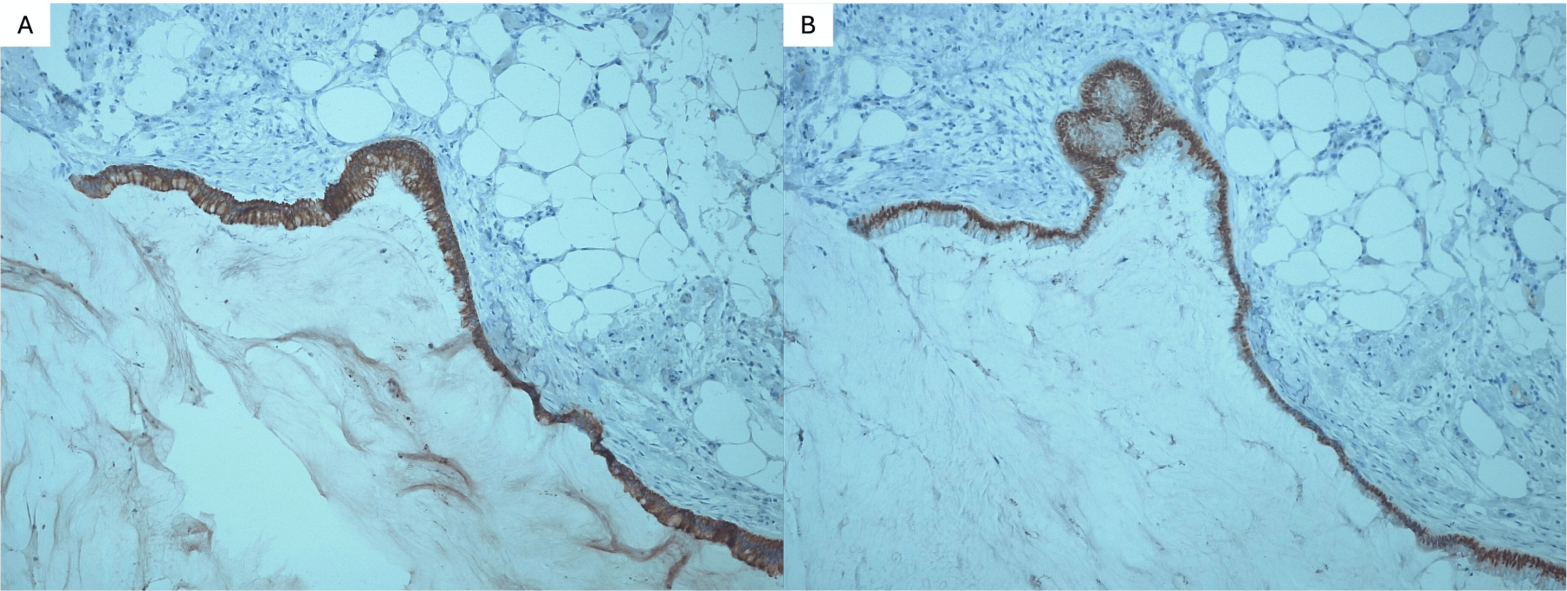
Immunohistochemical analysis of the omental sample. (A) CK20 positivity. (B) Positivity on CDX2

Immunohistochemical analysis of the omental sample revealed positivity for CK20 (Figure 2A) and CDX2 (Figure 2B), with negative expression of CK7 and PAX8.

During the postoperative Oncology Medical Advisory Board meeting, the patient was referred to a specialized board for gastrointestinal neoplasms for further management. It was recommended that she undergo cytoreductive surgery accompanied by hyperthermic intraperitoneal chemotherapy.

## Discussion

The simultaneous occurrence of a serous BOT, an LAMN accompanied by PMP, and endometrial cancer presents a challenge for both diagnosis and treatment. Each of these neoplasms has unique biological characteristics and requires a specific treatment approach. On the other hand, their simultaneous occurrence can complicate the clinical assessment, especially when mucinous peritoneal involvement raises questions about the source and spread of the peritoneal masses. To the best of our knowledge, this is the first case of synchronous serous BOT, endometrial adenocarcinoma, and LAMN with PMP.

PMP represents a distinct anatomoclinical entity marked by the deposition of neoplastic cells on peritoneal surfaces, leading to the excessive production of mucin [10]. The incidence of this rare condition is reported at 0.2 cases per 100,000 individuals annually, frequently identified as an incidental discovery through imaging studies or during surgical exploration [11]. The origin of PMP remains a contentious issue within the research community, with some scholars advocating for the ovaries as the primary site of origin, while others contend that the appendix vermiformis may be the source. Literature indicates the occurrence of pseudomyxoma peritonei originating from various organs, such as the colon and pancreas [12]. The World Health Organization categorized PMP as either low-grade or high-grade lesions, utilizing histological, molecular, and cytological characteristics for this classification [13]. Surgical debulking followed by hyperthermic intraperitoneal chemotherapy represents the fundamental approach to managing this peculiar condition [11].

LAMN occurs in fewer than 1% of appendectomy cases. The clinical outcomes of these neoplasms are often unpredictable, presenting significant challenges in diagnosis [6]. Individuals diagnosed with LAMN frequently present with symptoms reminiscent of appendicitis, including discomfort in the right iliac fossa, elevated temperature, nausea, and emesis [5]. It is noteworthy that nearly 25% of appendiceal mucinous neoplasms present without symptoms and are often discovered incidentally through abdominal imaging or surgical procedures [5]. The potential risk of these tumors progressing to PMP remains unquantified, creating a challenge regarding their subsequent management and surveillance strategies [14]. A recent study by Lakmal et al. demonstrated that nonperforated LAMN has a negligible risk of recurrence or developing PMP and that further surgery is not indicated in completely excised tumors [14]. Histological analysis of the appendiceal tissue in our case revealed a nonperforated LAMN and histologically healthy margins of the specimen.

A recent literature review about ovarian causes of PMP cited mucinous cystadenoma, mucinous ovarian cancer, malignant transformation of mature cystic teratoma, and mucinous BOT [7]. Furthermore, the authors indicated that appendiceal mucocele in women could mimic adnexal pathology, manifesting as a palpable pelvic mass. They concluded that identifying this condition can pose challenges before surgical intervention, whether through imaging techniques or during the operative stage. Literature suggests that the initial manifestations of the mentioned appendiceal condition may encompass nausea, vomiting, alterations in bowel patterns, gastrointestinal hemorrhage, and genitourinary symptoms [7]. In a case report of LAMN metastasis to the ovary, the authors offered possible guidelines for the intraoperative distinction between primary ovarian, primary appendiceal neoplasm, and features of metastasis to the ovary [15]. The authors highlighted that the primary ovarian neoplasms have smooth capsules and evenly distributed cystic and solid regions without nodularity. Mural nodules may be present in tumors of 10-13 cm in diameter. In contrast, metastatic ovarian cancer often presents as bilateral nodular ovarian masses. They further explained that the primary appendiceal neoplasms rarely involve the ovaries. Instead, they have a large mucinous multinodular appearance and often have PMP and extensive intraperitoneal dispersion. Finally, lesions or perforations suggest an appendix origin [15]. On the other hand, the relation between LAMN, PMP, and ovarian tumors almost exclusively suggests mucinous histology. Interestingly, BOT in our case had serous histological features.

Searching the literature, we found one case report of primary endometrioid endometrial cancer with synchronous LAMN and PMP [16]. In this case, the patient had a total laparoscopic hysterectomy with bilateral adnexectomy, pelvic lymphadenectomy, and appendicectomy.

## Conclusions

We presented a case of three different neoplasms in a single patient: endometrioid endometrial adenocarcinoma, serous BOT, and LAMN with PMP. According to our knowledge, this is the first such reported case in a literature. There are significant challenges in preoperative diagnosis of primary endometrial cancer accompanied by adnexal involvement, as well as the occurrence of synchronous neoplasms affecting both the endometrium and ovary. Moreover, many patients with LAMN and PMP may present with minimal symptoms. Therefore, it is essential to adequately prepare the patient for the potential need for an extended surgical procedure and to provide comprehensive information regarding the prognosis, which will be contingent upon the results of the histopathological analysis. Furthermore, it is our assertion that a collaborative approach between the oncological and surgical teams is essential when managing a patient with endometrial cancer and a unilocular adnexal mass, given the potential for unforeseen intraoperative findings, as illustrated in our case.
